# Nucleotide Supplementation to Whole Milk Has Beneficial Effects on Post-Weaning Holstein Calf Performance

**DOI:** 10.3390/ani11010218

**Published:** 2021-01-18

**Authors:** Yousef Abbaslou, Davood Zahmatkesh, Ehsan Mahjoubi, Mehdi Hossein Yazdi, Hamed Beiranvand, Morteza Gorjidooz

**Affiliations:** 1 Department of Animal Science, Faculty of Agriculture, University of Zanjan, Zanjan 45371-38791, Iran; y.abbaslo@znu.ac.ir; 2 Department of Animal Science, Faculty of Agriculture and Natural Resources, Arak University, Arak 38156, Iran; m-hosseinyazdi@araku.ac.ir; 3 Saffari-Salehi Agro-Industrial Co., Varamin, Tehran 33751-13111, Iran; hamedbeiran669@gmail.com; 4 Department of Clinical Science, Faculty of Veterinary Medicine, Islamic Azad University, Garmsar Branch, Garmsar 35816-311673581, Iran; gorjidoozm@yahoo.com

**Keywords:** calf, diarrhea, nucleotide, whole milk

## Abstract

**Simple Summary:**

The positive effects of nucleotide (NU) supplementation in milk replacer have been elucidated in infants and partly in dairy calves; however, NU addition to whole milk has not been evaluated yet. In this study, a NU-containing supplement was added to whole milk at rate of 0.5 and 1 g/d. Without any effects on pre-weaning performance, post-weaning intake was improved as NU increased and final body weight was greater in NU supplemented calves. Nucleotide supplementation to whole milk linearly decreased days with loose feces in the first month of life. These results show that NU has beneficial effect on calf health status and intake even if it is supplemented to whole milk.

**Abstract:**

The positive effects of nucleotide (NU) supplementation in milk replacer have been elucidated in infants and in dairy calves; however, NU addition to whole milk has not been evaluated previously. This study aimed to assess NU supplementation in the whole milk on calf growth and health. Thirty Holstein calves (body weight: 39.1 ± 1.0 kg; 3 d after birth) were randomly assigned to the following treatments: whole milk without any supplementation (NU0), whole milk + 0.5 g/d added a NU-containing supplement to whole milk (NUCS0.5), and whole milk + 1 g/d added a NU-containing supplement to whole milk (NUCS1). Calves were weaned at d 55 and stayed on study until d 75. Calves had free access to feed and water throughout the study. Dry matter intakes (DMI) were similar among treatments (*p* > 0.05) during the pre-weaning period; however, increasing NU resulted in a linear (*p* < 0.05) increase in DMI during the post weaning period (2158, 2432, and 2518 g/d for NU0, NUCS0.5, and NUCS1, respectively). Treatments did not affect body weight (BW) at the first and second month of study, but final BW linearly increased as NU was added (87.1, 90.6, and 95.4 kg for NU0, NUCS0.5, and NUCS1, respectively). Neither pre-weaning average daily gain nor post-weaning average daily gain was affected by treatments; accordingly, feed efficiency was similar among treatment groups. Days with loose fecal score were linearly decreased as NU was added to whole milk during the first month of life, while the fecal score did not differ among treatments until the end of the study. No difference was observed in the skeletal growth of calves in the current study. Therefore, it can be concluded that NU supplementation in the whole milk has some beneficial effects on calf performance in terms of final BW, post-weaning DMI, and less days with loose feces.

## 1. Introduction

Nucleotides are members of non-protein nitrogen compounds that are found in many foods such as seafood, legumes, and organ meats. Nucleotides supplementation in the diet of ruminants has attracted attention during the last years [1]. These are the functional ingredients that improve animal performance and their beneficial effects in animal health caused them to become required items in the diet of dairy cattle [1]. Nucleotides are especially important in the tissues with rapid cell proliferation (intestinal epithelial cells) and anywhere that the capacity of de novo pathway, which is the primary route of nucleotides production, is low. When the endogenous supply is insufficient, exogenous nucleotide sources tend to become semi-essential or “conditionally essential” nutrients [2]. This is true for infants and pre-weaning dairy calves [1,3].

Kehoe et al. [3] were one the first authors who included nucleotides in milk replacer for dairy calves; without any difference in calf growth or health, they concluded that nucleotides might have improved the intestinal environment. Besides, Chester-Jones et al. [4] showed that a slightly higher concentration of nucleotides in calf milk replacer (5%; ~22 g/d based on average fed milk replacer) decreased calf growth from 9 to 25 weeks of age. More recently, and using NU as a nitrogen source, Hill et al. [5] showed a linear decrease in calf average daily gain (ADG) and feed efficiency when nucleotides were added to milk replacer as much as 10 and 20% of DM (~50 and 100 g/d based on average fed milk replacer); they concluded that concentrations of ≥10% nucleotides could not be recommended for milk replacer in neonatal dairy calves.

All studies mentioned above, have been carried out with milk replacer, because dried milk products used in MR (Milk replacer) have low concentrations of nucleotides compared with whole milk and colostrum [3]. In humans, nucleotides have been included in infant formula, because it is believed that the biological advantages of breast milk over cow’s milk-based formulas are due to a higher concentration of nucleotides in breast milk [6]. If this is the case, it might worth adding NU to whole milk to obtain beneficial effects in dairy calves. To our knowledge, there is no study in neonatal calves in which NU has been added to whole milk. Therefore, our primary goal was to evaluate the potential effects of NU supplementation to milk for pre-weaning calves. We hypothesized that the addition of NU to whole milk could improve calf growth and health.

## 2. Materials and Methods

### 2.1. Calves, Housing, and Feeding

**Figure 1:**
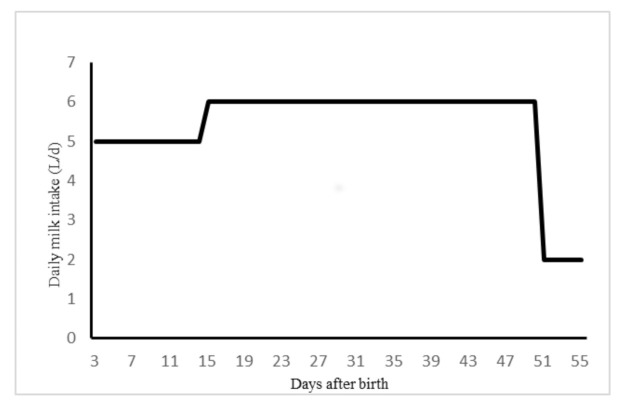
Milk feeding method for calves (5 L of milk/d from 3 to 14 d, 6 L/d from 15 to 50 d, and 2 L/d from 51 to 55 d of age).

All experimental procedures conducted in this study were in accordance with the guidelines of the Iranian Council of Animal Care (1995; #19356) [7]. This experiment was conducted in a commercial dairy farm (Agro-Industrial Co., Varamin, Tehran, Iran) during the summer and fall of 2019. This farm is located in a tropical area (35°19′ N 51°41′ E). After birth, 30 Holstein male and female calves (body weight = 39.1 ± 1.0 kg) received colostrum (at least 4 L within the first 4 h after birth and the second meal in 12 h; Brix% of 20–22) and were enrolled in the study within 72 h of birth in a complete randomized block design. Calves received 5 L/d whole milk (3.42 ± 0.13% fat, 3.14 ± 0.08% CP(Crude protein), 4.66 ± 0.04% lactose, and 12.01 ± 0.14% total solids) in steel buckets twice a day at 0900 and 1600 h from d 3 to 14; thereafter, they received 6 L/d from d 15 to 50 of the study, and then 2 L/d and only one meal from d 51 to 55 at 0900 h (Figure 1). On d 2 of life, calves received transition milk and from d 3 onwards, calves were individually fed whole milk. All calves were weaned at 55 days of age and stayed in the study until d 75.

Experimental treatments were: control without NU supplementation (NU0; *n* = 5 males and 5 females); 0.5 g/d NU [Ascogen^®^ (dry matter: 91.8%; Ash: 7.3%; crude protein: 48.5%; ether extract: 1.4%; crude fiber: 0.3%, total nucleotide content >14%); Chemoforma, Switzerland; https://nuproxa.ch/products/ascogen/] was added to milk from d 3 to 55 (NUCS0.5; *n* = 5 males and 5 females); 1 g/d NU was added to milk (NUCS1; *n* = 5 males and 5 females). Calves were housed in individual pens (1.5 × 2.5 m^2^) bedded with straw and treatments were completely randomly assigned (10 calves/treatment). All calves had free access to fresh water and starter feed was offered ad libitum since the beginning of the experiment. The calves received the starter feed mixed with 100 g/kg DM chopped wheat straw as a total mixed ration (TMR). Fresh feed was offered every morning at 0800 h. Diet was formulated using software available from the NRC (National research council) [8]. The ingredients and nutrients composition of the basal diet are shown in Table 1.

### 2.2. Sample Collection, and Measurements

Individual starter feed intake was determined from the daily offered and refused amount. Calves were weighed seven times during the experiment (10-d intervals; using an electronic scale), beginning from the commencement of trial; wither and hip height were measured also on the same days. Feed efficiency (kg of BW gain/kg of total DMI) was calculated accordingly. Samples from feeds and orts were collected and the subsamples of feeds and refusals were mixed thoroughly, dried (analysis of DM: drying sample in an oven at 105 °C for 24 h, method 925.40; AOAC, 1990) [9], and ground to pass a 1-mm screen in a mill (Ogawa Seiki CO., Ltd., Tokyo, Japan) before chemical analysis for CP (method 988.05; AOAC, 1990) [9] and lipid (method 920.39; AOAC, 1990) [9].

Fecal scores were daily obtained by farm technician (the same person throughout study who was not aware of treatments) on each calf using a scale of one to four with the following definitions: 1 = firm, 2 = soft, 3 = soft and running, and 4 = watery [10]. For calves which need medical treatments, farm’s veterinarian administrated the proper drug and the treatment was followed according to his recommendation; therefore, medical days, number of used drugs, treatment bouts and fluid therapy were recorded to be statistically analyzed.

### 2.3. Statistical Analysis

Prior to data analysis, normality of the continuous data was checked using UNIVARIATE Proc in SAS (SAS version 9.1; SAS Inst. Inc. Cary NC). All data (DMI, BW, skeletal growth, and health criteria) were subjected to an analysis of variance using a mixed model for repeated measures. The final statistical model included the fixed effects of treatment, sex, time, and time × treatment interaction; and calf was considered as the random effect. Sex was not significant and removed from the model. Body weight and body skeletal growth data were not analyzed as repeated measure and they were analyzed by the GLM (General linear model) with the fixed effects of treatment and sex; sex was again removed from the final model because it was not significant. Data were analyzed using polynomial contrasts to evaluate for linear effects of NU addition. The covariance structure that yielded the smallest Akaike’s information criterion was used. The BW data on d 3 was considered as a covariate for analysis.

Diarrhea data was categorized in the number of days with fecal score ≥ 3 [11]. The variance of fecal scores was not uniformly distributed. Therefore, fecal scores were square-root transformed for better homogeneity of the distribution of residuals. The same was done for medical days, number of used drugs, treatment bouts, and fluid therapy. Because of more prevalence of diarrhea within the first month of life, relevant data were subdivided to d 3–30, d 31–55, and d 3–75. Least squares means for treatment effects were separated by the use of the PDIFF statement when the overall *F*-test was *p* ≤ 0.05. Trends were declared when 0.05 < *p* ≤ 0.10.

## 3. Results

As indicated in Table 2, there were no differences in initial BW, wither or hip height among experimental treatments. Although the time effect was significant and DMI increased with the advancement in the study, total DMI (milk DM + starter DM) during pre-weaning was similar among treatments. Nevertheless, a tendency for the day by treatment interaction (*p* = 0.10) showed that NU supplemented calves more rapidly increased starter intake compared with the control group. Accordingly, pre-weaning ADG and BW at d 30 and 55 as well as feed efficiency were not different for experimental groups. Treatments had no effect on post-weaning ADG and feed efficiency, but starter intake was linearly increased as NU was supplemented (*p* < 0.04); because of that, the final BW linearly improved in NU supplemented calves (*p* < 0.02). Similar to ADG measures, hip and wither heights were not affected by treatment during the pre- and post-weaning periods.

Days with loose fecal score were linearly decreased as NU was added to whole milk during the first month of life (*p* = 0.05), while the fecal score did not differ among treatments until the end of the study (Table 3).

## 4. Discussion

In contrast to our hypothesis, DMI and growth performance were not affected by NU supplementation during the pre-weaning period. Although Kehoe et al. [3] did not observe any difference in feed intake and efficiency among treatments over a 6-week period, they reported that the control group tended to have less starter intake during the 6th week compared with the yeast-derived NU supplemented calves. Similar to the current study, rats fed a regular diet without extra supplementation compared with rats fed dietary supplemented-nucleotides showed no declined growth rates [12]. In malnourished children, nucleotide intake was shown to enhance growth in weight, length, and head circumference in infants born small [13] and to increase biomarkers that could influence catch-up growth [14]. Hill et al. [5] also found no effect of NU in milk replacer on pre-weaning DMI. In contrast with the current study, however, Hill et al. [5] reported the decreased pre-weaning ADG and feed efficiency, probably because NU was supplemented at a very high dosage and as an N source in milk replacer. Unexpectedly, post-weaning starter intake linearly increased in NU supplemented groups without any change in ADG or feed efficiency. Kehoe et al. [3] and Hill et al. [5] indicated that post-weaning feed intake was not affected by nucleotide supplementation. Considering the greater tendency of NU fed calves to eat more starter feed when they approached to weaning, it appears that the beneficial effect of NU on starter intake has postponed to the post-weaning period. Wood et al. [15] suggested that weaning may disrupt the permeability of the GIT (Gastrointestinal tract). On the other hand, it has been proposed that NU can improve intestinal epithelium repair, gut development [16,17] and intestinal environment [3]. Therefore, it appears that the intestine environment has been improved and, in turn, has led to better intake during post-weaning period. Recently, Adab et al. [18] also showed that Zn-glycine (which has been shown to improve small intestinal integrity) results in increased DMI around weaning and post-weaning period. Because of the greater post-weaning DMI, the heavier final BW in NU fed calves was not surprising. Król [19] also showed higher final BW in calves fed yeast nucleotides in milk replacer, which is in line with the current results.

The day with fecal score ≥ 3 tended to be linearly (*p* = 0.10) decreased with NU supplement throughout the study; this was mostly because of the reduced days with diarrhea during the first month of life (*p* < 0.05). The diarrhea is one of the most causes of mortality and morbidity in pre-weaning period (which can cause the growth performance to be disrupted; [20]) and it has been found that NU improve intestinal maturation [21] and aid recovery from diarrhea [22,23].

Fewer days with the abnormal scores in NU-fed calves could confirm their better health condition. In line with the current study, days with abnormal fecal score was linearly decreased when NU was supplemented in the Hill et al. [5] study during the pre-weaning period and throughout the study; the reductions in days with abnormal fecal scores were attributed to poor digestion of NU, which might have increase fecal output of solids. Similar to our results, Król [19] observed that the fecal score was worse in calves fed yeast nucleotides in milk replacer during the first month of life. Fecal scores were not influenced by treatment in another study [3], probably because of low dosage and the source of NU (purified nucleotides or nucleotides from an extract of *Saccharomyces cerevisiae*); however, in that study, treatment by week interaction revealed that the control group (calves without any additive) had higher fecal scores during weeks 2, 3, and 4 compared with NU-treated calves.

In line with the current results, days with medical treatments (Table 3) did not differ when NU was fed at 0, 10, or 20% of milk replacer DM [5]. It was expected that days with medical treatments would decrease, because days with loose feces had reduced. It has been shown that dietary NU can affect immune function and may have beneficial effects on gastrointestinal tract growth and maturation [24], probably affecting medical treatment days. Jiao et al. [25] showed that sows receiving nucleotides had increased fecal *Lactobacillus* counts and decreased *Escherichia coli* counts at weaning day; however, they found no difference in fecal score and diarrhea in piglets and concluded that the nucleotides could influence intestinal health and have positive effects on excreta microflora in sows at weaning day without any impact on medical treatment days.

Although the number of drug used and treatment bouts were not different among groups (Table 3), in the control group, 4 heads out of 10 calves received fluid therapy during the pre-weaning period while only 2 out of 10 calves in each NU treated group were subjected to fluid therapy (Table 3). This is very important, based on the cost of current therapy and the long-term effect of the therapy early in life on future productive performance of calves. Heinrichs and Heinrichs [26] concluded that days of illness and days treated before 4 months had significant effects on the first-lactation production of Holsteins.

Hip width linearly decreased when NU was increased in milk replacer [5]; less feed intake was the main cause of declined hip width in the study of Hill et al. [5]. Furthermore, hip and withers heights were not affected by treatments in other study [3]. It appears that the NU effects on skeletal growth measurements is minor and the principal place on which NU has some effects is the small intestine, as it was observed in fewer days with abnormal fecal scores.

## 5. Conclusions

In conclusion, for the first time, the potential effects of NU supplementation into the whole milk on dairy calves’ performance have been evaluated in the current study. The NU addition to whole milk did not affect DMI during the pre-weaning period; although NU was supplemented only in whole milk, the supplementation resulted in a linear increase in DMI during the post-weaning period. Final BW linearly increased as NU was increased while there was no difference in the pre-weaning BW gain. Loose feces were linearly decreased as NU was added to whole milk during the first month of life. Generally, it can be concluded that NU supplementation into the whole milk has some beneficial effects on the productivity and the health of calves.

## Figures and Tables

**Table 1:** Description of starter diet.

Items	Values
Ingredients, g kg/DM	
Wheat straw	100
Corn grain, meal	502
Wheat bran	45
Soybean meal, 45% CP	288
Sodium bicarbonate	9
Calcium carbonate	18
Salt	9
Bentonite	14
Vitamin and mineral mix ^1^	15
Nutrient composition	
DM, g/kg	913
Crude protein, g/kg DM	181
Metabolisable energy, MJ/kg ^2^	10.5
Net energy for maintenance, MJ/kg ^2^	6.0
Net energy for gain, MJ/kg ^2^	1.44
Neutral detergent fiber, g/kg	249
Ether extract, g/kg DM	29

^1^ Contained per kg of premix: 250,000 IU of vitamin A, 50,000 IU of vitamin D, 1500 IU of vitamin E, 2.25 g of Mn, 120 g of Ca, 7.7 g of Zn, 20 g of P, 20.5 g of Mg, 186 g of Na, 1.25 g of Fe, 3 g of S, 14 mg of Co, 1.25 g of Cu, 56 mg of I, and 10 mg of Se. ^2^ Calculated using NRC (2001) model.

**Table 2:** Effects of nucleotide (NU) supplementation to whole milk on productive performance of Holstein calves.

	Treatment ^1^			SEM	*p*-Value			
	NU0	NUCS0.5	NUCS1		Treatment	Time	Time × Treatment	Linear
BW, kg								
d 3 (Initial)	39.1	39.3	38.8	1.03	0.83	-	-	0.84
d 55 (Weaning)	68.2	68.8	71.1	1.76	0.53	-	-	0.26
d 75 (Final)	87.1	90.6	95.4	2.21	0.12	-	-	0.02
DMI, g DM/d								
Pre-weaning	1142	1237	1259	59.2	0.35	0.01	0.16	0.17
Post-weaning	2728	2965	3154	141	0.12	0.01	0.19	0.04
Average daily gain, g/d								
Pre-weaning	658	694	726	36.6	0.43	0.01	0.33	0.53
Post-weaning	1120	1114	1260	87.6	0.48	0.01	042	0.17
Gain:feed ratio ^2^								
Pre-weaning	0.59	0.58	0.60	0.02	0.73	0.01	0.44	0.82
Post-weaning	0.41	0.38	0.40	0.02	0.76	0.01	0.63	0.66
Withers height, cm								
d 3 (Initial)	77.4	77.4	77.3	0.83	0.99	-	-	0.93
d 55 (Weaning)	86.9	88.0	87.5	0.66	0.94	-	-	0.52
d 75 (Final)	90.2	91.1	91.0	0.56	0.51	-	-	0.35
Hip height, cm								
d 3 (initial)	79.9	80.3	79.8	0.82	0.53	-	-	0.36
d 55 (Weaning)	90.2	91.4	90.6	0.64	0.77	-	-	0.89
d 75 (Final)	93.6	94.5	94.4	0.58	0.53	-	-	0.37

^1^ Treatments: (1) NU0 = without nucleotide (NU) supplement; (2) NUCS0.5 = 0.5 g/d NU supplement added to milk; (3) NUCS1 = 1 g/d NU supplement added to milk. ^2^ kg of body weight gain/dry matter intake.

**Table 3:** Effects of nucleotide (NU) supplementation to whole milk on health of Holstein calves.

	Treatment ^1^			SEM	Linear Effect
	NU0	NUCS0.5	NUCS1		
Fecal score ^2^					
d 3-30	1.60	1.19	0.29	0.06	0.05
d 31-55	0.01	0.06	0.01	0.01	0.53
d 3-75 (throughout study)	1.60	1.37	0.41	0.07	0.10
Medical days ^3^	1.30	0.91	1.29	0.49	1.00
Fluid therapy, day	0.40	0.20	0.20	0.14	0.33
Number of used drugs ^4^	1.40	0.90	1.19	0.50	0.78
Treatment bouts	0.50	0.30	0.50	0.18	1.00

^1^ Treatments: (1) NU0 = without nucleotide (NU) supplement; (2) NUCS0.5 = 0.5 g/d NU supplement added to milk; (3) NUCS1 = 1 g/d NU supplement added to milk. ^2^ Days with fecal score ≥3 (where 1 = firm, 2 = soft, 3 = soft and running, and 4 = watery). ^3^ Treatment was carried out under on-farm protocol and according to farm’s veterinarian. ^4^ Depending on the circumstances, the used drugs were: Ketprolak^®^ (Ketoprofen 10%; Bayer Aflak, Pharmaceutical Co, Lorestan, Iran); FlumaxM^®^ (Flunixin Meglumine 5%; Rooyan Darou, Tehran, Iran); Meloxicam^®^ (Meloxicam 20 mg/mL; Rooyan Darou, Tehran, Iran); B Co ject^®^ (B Complex, Rooyan Darou, Tehran, Iran); F-nex^®^ 300 (Florfenicol 300 mg; Razak Laboratories, Karaj, Iran); Enroflak^®^ 10% (Enrofloxacin; Bayer Aflak, Pharmaceutical Co, Lorestan, Iran).
